# Risk factors for longitudinal changes in left ventricular diastolic function among women and men

**DOI:** 10.1136/heartjnl-2018-314487

**Published:** 2019-04-01

**Authors:** Oscar L Rueda-Ochoa, Marco A Smiderle-Gelain, Dimitris Rizopoulos, Klodian Dhana, Jan-Kees van den Berge, Luis E Echeverria, M Arfan Ikram, Jaap W Deckers, Oscar H Franco, Maryam Kavousi

**Affiliations:** 1 Department of Epidemiology, Erasmus MC, Rotterdam, The Netherlands; 2 Department of Basic Sciences, Universidad Industrial de Santander, Bucaramanga, Colombia; 3 Department of Cardiology, Federal University of Health Sciences UFCSPA, Porto Alegre, Porto Alegre, Brazil; 4 Department of Biostatistics, Erasmus Medical Center, Rotterdam, Netherlands; 5 Heart Failure and Heart Transplant Clinic, Fundacion Cardiovascular de Colombia, Floridablanca, Colombia; 6 Department of Cardiology, Erasmus MC, Rotterdam, The Netherlands

**Keywords:** echocardiography, doppler, left ventricular diastolic function, cohort study, sex difference, risk factors

## Abstract

**Objective:**

To evaluate changes in left ventricular diastolic function (LVDF) parameters and their associated risk factors over a period of 11 years among community-dwelling women and men.

**Methods:**

Echocardiography was performed three times among 870 women and 630 men (age 67±3 years) from the prospective population-based Rotterdam Study during a period of 11-year follow-up. Changes in six continuous LVDF parameters were correlated with cardiovascular risk factors using a linear-mixed effect model (LMM).

**Results:**

In women, smoking was associated with deleterious longitudinal changes in deceleration time (DT) (Beta (β): 7.73; 95% CI 2.56 to 12.9) and high-density lipoprotein cholesterol was associated with improvement of septal e′ (β: 0.37; 95% CI 0.13 to 0.62) and E/e′ ratio (β: −0.46; 95% CI −0.84 to –0.08) trajectories. Among men, diabetes was associated with deleterious longitudinal changes in A wave (β: 3.83; 95% CI 0.06 to 7.60), septal e′ (β: −0.40; 95% CI −0.70 to –0.09) and E/e′ ratio (β: 0.60; 95% CI 0.14 to 1.06) and body mass index was associated with deleterious longitudinal changes in A wave (β: 1.25; 95% CI 0.84 to 1.66), E/A ratio (β: −0.007; 95% CI −0.01 to –0.003), DT (β: 0.86; 95% CI 0.017 to 1.71) and E/e′ ratio (β: 0.12; 95% CI 0.06 to 0.19).

**Conclusions:**

Smoking among women and metabolic factors (diabetes mellitus and body mass index) among men showed larger deleterious associations with longitudinal changes in LVDF parameters. The favourable association of HDL was mainly observed among women. This study, for the first time, evaluates risk factors associated with changes over time in continuous LVDF parameters among women and men and generates new hypothesis for further medical research.

## Introduction

Left ventricular diastolic dysfunction is highly prevalent and worsen with advancing age.^1–3^ Persistence or progression of diastolic dysfunction is a risk factor for heart failure (HF) among the elderly.^2^ Recent data suggest that diastolic dysfunction is present in the majority, around 70% of patients with heart failure with preserved ejection fraction (HFpEF).^4^ Although plenty of evidence-based treatments for heart failure with reduced ejection fraction exist, there is no treatment with proven benefits for HFpEF.^5^

Impairment of left ventricular diastolic function (LVDF) occurs gradually and has been shown to be, at least partly, reversible.^1 6^ Therefore, early detection of subclinical impairment in LVDF and identification and treatment of its associated risk factors to prevent or slow the progression to overt HF is important. To date, several risk factors associated with LVDF have been identified.^7 8^ However, longitudinal studies evaluating changes in continuous LVDF parameters over time in a general population of subjects without clinically diagnosed HF are scant and have been mostly performed among middle-aged individuals. As the occurrence of various HF phenotypes differs between women and men,^5^ it has been suggested that gender differences in susceptibility to risk factors might partly explain these dissimilarities.^6^ However, recent studies have failed to address gender differences in the set of changes in LVDF and its associated risk factors.^4–7^ Notably, while women tend to have a better LVDF until 60 years of age, gender disparities are reversed after the menopause.^5^ To further clarify sex differences in the pathophysiology of diastolic dysfunction, studying changes in continuous LVDF parameters among women and men and their correlates, especially at older ages, is warranted.

We, therefore, aimed to evaluate longitudinal changes in continuous LVDF parameters during 11 years of follow-up among women and men from a large prospective population-based cohort.^9^ Participants were all free from clinically diagnosed HF at the time of echocardiographic examinations and during follow-up. In addition, we investigated the risk factors associated with the changes in LVDF parameters among women and men.

## Methods

### Study population

The Rotterdam Study (RS) is a prospective population-based cohort that included participants aged ≥55 years in the district of Ommoord, Rotterdam, The Netherlands.^9^ The study started in 1990 with 7983 participants (RS-I) and was extended twice: in 2000 (RS-II, n=3014) and 2006 (RS-III, n=3932). The follow-up examinations take place every 3–4 years. The RS was approved by the Medical Ethics Committee according to the Population Study Act Rotterdam Study. All participants provided written informed consent.

**Figure 1:**
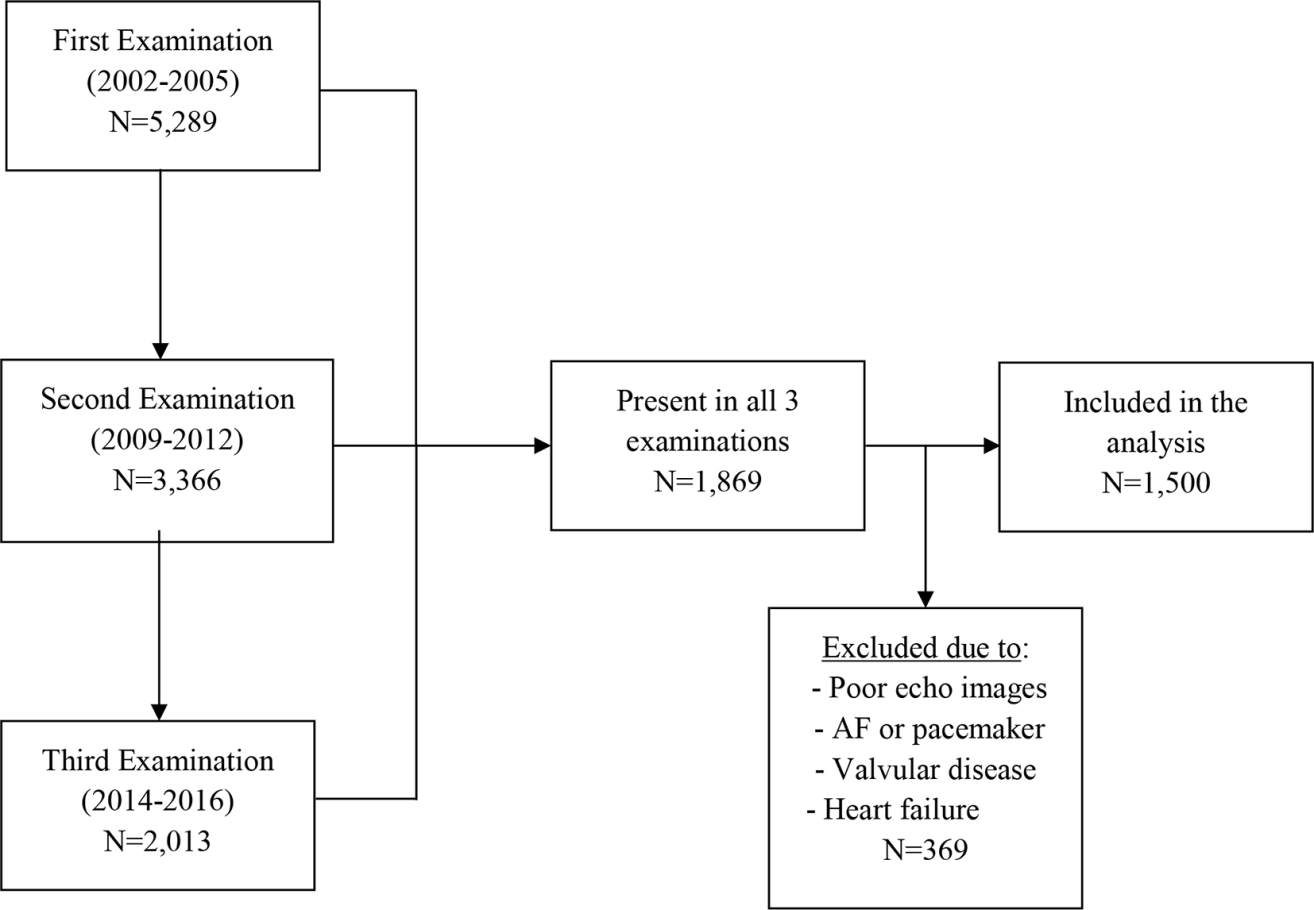
Flowchart for the participants included in the analysis of longitudinal changes in LVDF parameters measured 3 times over 11 years of follow-up. AF, atrial fibrillation; LVDF, left ventricular diastolic function.

The present study used data for six LVDF echocardiographic parameters from the fourth, fifth and sixth examinations of the first cohort (RS-I) and the second, third and fourth examinations of the second cohort (RS-II). Out of the six LVDF parameters under study, three repeated echocardiographic measurements were available for four indexes among 1869 participants. We excluded 369 individuals due to poor echocardiographic images, atrial fibrillation, artificial pacemaker, moderate–severe valve compromise and clinically diagnosed HF at the time of echocardiographic examinations and during the follow-up. Therefore, we included a total of 1500 participants (630 men and 870 women) (figure 1). For two LVDF parameters, two repeated measurements were available in a total of 1528 (646 men and 882 women) subjects from the fifth and sixth examinations of the first cohort (RS-I) and the third and fourth examinations of the second cohort (RS-II) (online supplementary figure 1).

### LVDF parameters

We studied six continuous LVDF parameters: the early transmitral ventricular diastolic filling velocity (E wave), late diastolic filling velocity (A wave) and the early diastolic longitudinal filling velocity of the septal mitral annulus (septal e′) during three cardiac cycles. The means of the E wave, A wave and septal e’ were used to calculate E/A and E/e′ ratios. Mitral valve deceleration time (DT) was measured as the time between the peak E-top wave and the upper deceleration slope extrapolated to the zero baseline using a continuous wave Doppler.^10 11^ Additional information on echocardiographic measurements is provided in the online-supplementary material.

10.1136/heartjnl-2018-314487.supp1 Supplementary file 1

### Assessment of cardiovascular risk factors

The detailed information regarding the evaluation of cardiovascular risk factors is given in the online supplementary material.

## Statistical analysis

In the descriptive analysis, continuous variables with normal distribution were reported as mean (SD) and categorical variables as numbers (percentages). We compared the mean and percentage values for women and men using t-test and z-proportion tests, respectively. Longitudinal changes in LVDF parameters over time were plotted, treating age as a time-varying covariate. For each of the six parameters, a longitudinal data analysis using an LLM was performed. The outcome of interest in each model was the two or three repeated measurements for each index as a continuous variable. Age (as a time-varying covariate), systolic and diastolic blood pressure (SBP, DBP), heart rate (HR), total and high-density lipoprotein (HDL) cholesterol, blood pressure-lowering medication, lipid-lowering medication (LLM), diabetes mellitus (DM), current smoking, coronary heart disease (CHD), left ventricular mass (LVM) indexed by body surface area, left ventricular ejection fraction (LVEF), physical activity, left atrial diameter (LAD) and cohort were included in the fixed part of all models. The random part of the models only included age (as a time-varying covariate). All analyses were performed in total population and women and men separately. We checked for possible interaction between sex and different covariates in the total population. We additionally checked for the interaction terms between age, as a time-varying, and all covariates. We also compared the characteristics of the included participants with those who did not return for the follow-up echocardiography examinations. For more details regarding the analyses, consult the online supplementary material. The analyses were performed with R V.3.2.5 (R Foundation for Statistical Computing, Vienna, Austria), and STATA V.14.0 (Stata Corp, College Station, TX). A two-sided p value of <0.05 was considered statistically significant. Additionally, we considered a more conservative Bonferroni-corrected p value of <0.0083 (=0.05/6, considering six LVDF parameters).

## Results

Table 1 details the baseline characteristics of 870 women and 630 men for the analyses of E wave, A wave, E/A ratio and DT, in whom three repeated measurements were available during 11.1 years of follow-up. Women had higher HR, total and HDL cholesterol, LAD and ejection fraction. Men had larger DBP, LVM, left ventricular end-diastolic diameter (LVEDD), and left ventricular end-systolic diameter (LVESD) and CHD prevalence. For septal e′ and E/e′ ratio, two repeated measurements were available among 882 women and 646 men during 4.2 years of follow-up (online supplementary table 1).

**Table 1:** Baseline clinical and echocardiographic characteristics of the participants

	Women (n=870)	Men (n=630)	P value*
Clinical features			
Age, years	67.30 (4.95)	67.29 (4.91)	0.980
BMI, kg/m²	27.42 (4.07)	27.08 (2.94)	0.069
SBP, mm Hg	144.40 (18.32)	143.92 (19.20)	0.626
DBP, mm Hg	79.84 (10.11)	82.01 (9.90)	<0.001
Blood pressure-lowering medication, n (%)	261 (30.0)	208 (33.0)	0.2130
Hypertension, n (%)	609 (70.0)	446 (70.8)	0.7378
Heart rate, beats/min	69.36 (9.70)	65.79 (10.55)	<0.001
Total cholesterol, mmol/L	5.96 (0.94)	5.45 (0.93)	<0.001
HDL cholesterol, mmol/L	1.60 (0.40)	1.31 (0.31)	<0.001
Lipid-lowering medication, n (%)	174 (20.0)	130 (20.63)	0.765
Current smoker, n (%)	106 (12.2)	58 (9.2)	0.069
Prevalent CHD, n (%)	16 (1.84)	61 (9.68)	<0.001
Prevalent DM, n (%)	84 (9.66)	62 (9.84)	0.908
Echocardiography features			
LVM index, g/m²	70.66 (15.47)	78.17 (18.19)	<0.001
Left atrium diameter/BSA, mm/m²	21.41 (2.69)	20.76 (2.45)	<0.001
LVEDD, mm	49.39 (4.96)	53.36 (4.86)	<0.001
LVESD, mm	30.12 (7.87)	33.66 (8.01)	<0.001
Relative wall thickness, cm	0.29 (0.06)	0.29 (0.05)	1
Ejection fraction, %	65.87 (6.75)	63.69 (7.92)	<0.001
E wave, cm/s	67.38 (13.02)	64.48 (12.97)	<0.001
A wave, cm/s	83.33 (17.82)	76.61 (17.68)	<0.001
E/A ratio	0.83 (0.18)	0.86 (0.20)	<0.001
Deceleration time	204.4 (35.54)	209.19 (39.78)	<0.001
e`septal	6.87 (1.79)	7.29 (1.78)	<0.001
E/e`septal ratio	10.43 (2.62)	9.54 (2.51)	<0.001

Values are mean (±SD) or numbers (percentages).

*P value for comparison of different characteristics between women and men.

BMI, body mass index; BSA, body surface area; CHD, coronary heart disease, DBP, diastolic blood pressure; DM, type 2 diabetes mellitus; LVEDD, left ventricle end diastolic dimension; LVESD, left ventricle end systolic dimension; LVM, left ventricular mass; SBP, systolic blood pressure.

### Longitudinal changes in LVDF among women and men

**Figure 2:**
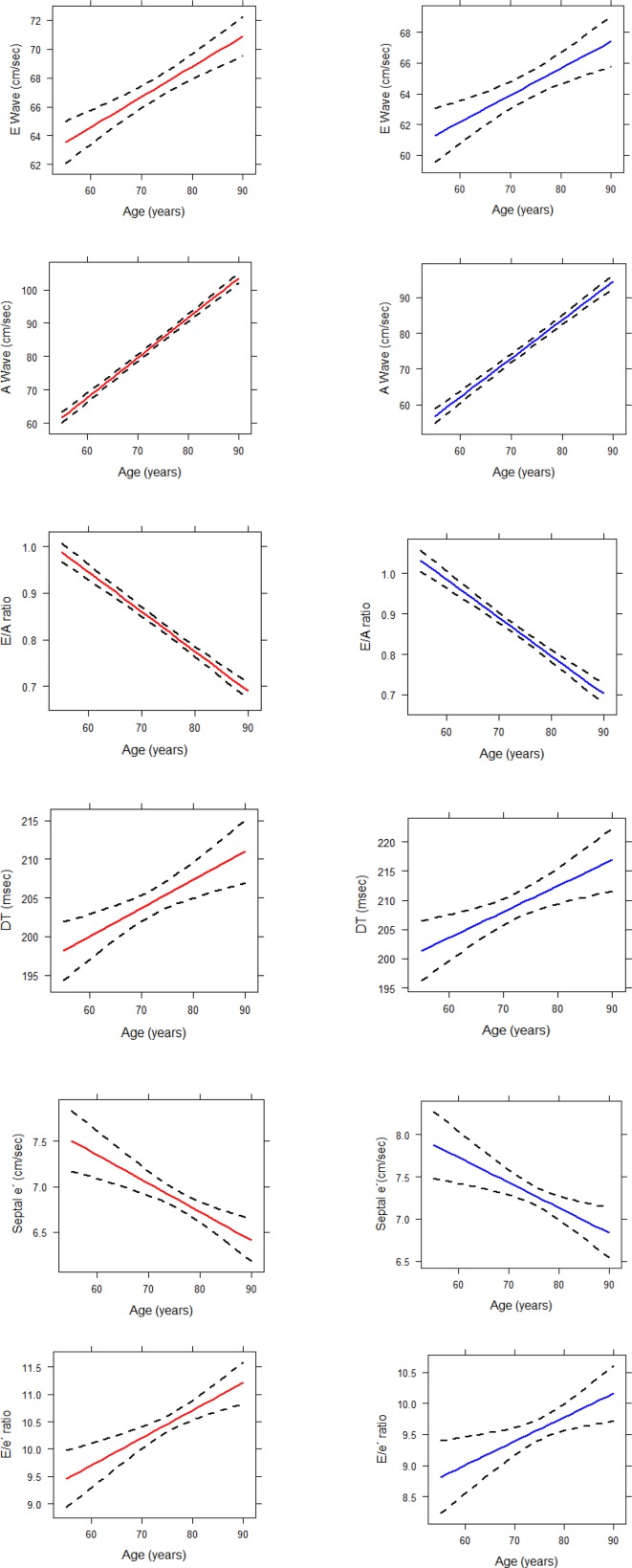
Plots for changes in LVDF parameters over time among women and men (red line: women, blue line: men). LVDF, left ventricular diastolic function.

Longitudinal changes in LVDF parameters were plotted against age (as a time-varying covariate), based on univariate models. The shapes of the longitudinal changes in all six LVDF parameters over time were similar in women and men (figure 2). There was no interaction between age (as a time-varying covariate) and sex. The plots revealed a progressive deleterious mono-directional change in the longitudinal trajectories of all six LVDF parameters over time; that is, a gradual rise in E wave, A wave, DT and E/e´ ratio values and a gradual decline in E/A ratio and septal e′. Despite similar trends in LVDF changes in both sexes, there were statistically significant differences in the mean values, with overall poorer indexes in women. The online supplementary table 2 presents detailed information on cross-sectional values for LVDF parameters per age and gender category.

### Risk factors associated with longitudinal changes in LVDF

As E wave, A wave, DT and E/e´ ratio values progressively, and deleteriously, raised over time, a positive beta coefficient for a risk factor means that the risk factor was associated with an increment in the trajectory of these LVDF parameters over time. On the contrary, a negative beta coefficient means that the risk factor was associated with a decrement in the trajectory of these LVDF parameters over time. Therefore, a positive risk factor coefficient is associated with an unfavourable progression and a negative risk factor coefficient into a favourable progression on LVDF parameters over time. E/A ratio and septal e′ values progressively, and deleteriously, diminish over time. Therefore, a negative beta coefficient for a risk factor means that the risk factors were associated with decrement and a positive coefficient means that the risk factor was associated with an increment in the trajectory of these LVDF parameters over time. Therefore, a positive coefficient translates into a favourable progression and a negative coefficient into an unfavourable progression on LVDF parameters over time.

**Figure 3:**
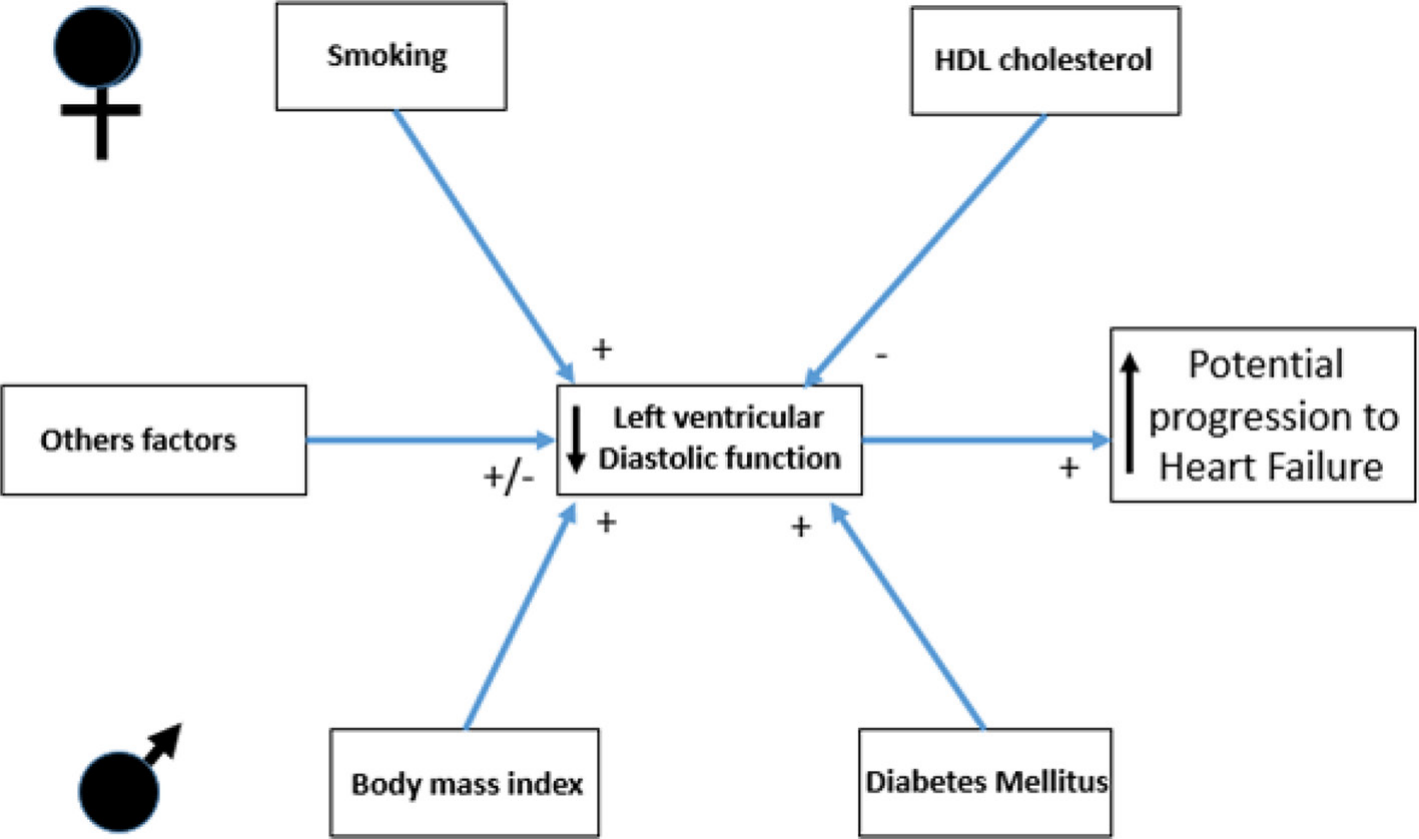
The core findings of our study, showing the main risk factors associated with longitudinal changes in LVDF parameters among women and men. LVDF, left ventricular diastolic function.

Tables 2 and 3 show all beta coefficients and CIs of different risk factors with longitudinal changes in LVDF indexes over time among women and men. Online supplementary tables 3 and 4 show the summary of the risk factors significantly associated with longitudinal changes in LVDF parameters among women and men. Figure 3 shows the core findings of our study, summarising the main differences among women and men in risk factors associated with changes in LVDF trajectories.

**Table 2:** Association of risk factors with longitudinal changes in LVDF parameters among women

	E Wave	A Wave	E/A ratio	DT	Septal e′	E/e′ ratio
Age*	4.43 (2.32 to 6.53) †	1.22 (1.14 to 1.30) †	−0.43 (−0.47 to –0.38)†	0.44 (0.20 to 0.68)†	−0.02 (−0.005 to –0.04)‡	0.015 (−0.05 to 0.019)
BMI	0.24 (0.04 to 0.44)‡	0.51 (0.25 to 0.76)†	−0.0008 (−0.003 to 0.002)	0.015 (−0.45 to 0.48)	0.02 (−0.002 to 0.05)	−0.015 (−0.06 to 0.03)
SBP	0.11 (0.06 to 0.17)†	0.18 (0.11 to 0.24)†	−0.0002 (−0.0008 to 0.0005)	−0.15 (−0.27 to –0.03)‡	−0.006 (−0.01 to 0.0009)	0.011 (−0.0005 to 0.02)
DBP	−0.20 (−0.29 to –0.11)†	−0.10 (−0.22 to 0.01)	−0.002†(−0.003 to –0.0006)	0.10 (−0.11 to 0.31)	−0.001 (−0.01 to 0.01)	−0.007 (−0.03 to 0.01)
BP-lowering medication	−1.63 (−3.40 to 0.15)	−0.12 (−2.39 to 2.16)	−0.01 (−0.03 to 0.01)	1.77 (−2.38 to 5.91)	−0.22 (−0.44 to 0.007)	0.27 (−0.074 to 0.61)
Heart rate	−0.02 (−0.10 to 0.06)	0.37 (0.26 to 0.47)†	−0.003† (−0.004 to –0.002)	−0.18 (−0.37 to 0.005)	−0.01 (−0.02 to 0.0006)	0.014 (−0.003 to 0.03)
Total cholesterol	0.009 (−0.80 to 0.83)	−0.14 (−1.17 to 0.89)	−0.0004 (−0.01 to 0.01)	−0.62 (−2.51 to 1.27)	−0.07 (−0.18 to 0.032)	0.06 (−0.10 to 0.23)
HDL cholesterol	1.19 (−0.73 to 3.10)	−1.12 (−3.51 to 1.27)	0.016 (−0.008 to 0.04)	−3.70 (−8.07 to 0.67)	0.37 (0.13 to 0.62)†	−0.46 (−0.84 to –0.08)‡
Lipid-lowering medication	0.58 (−1.35 to 2.50)	2.03 (−0.44 to 4.49)	−0.001 (−0.03 to 0.01)	−1.51 (−5.96 to 2.95)	−0.28 (−0.54 to –0.03)‡	0.55 (0.15 to 0.96)†
Current smoking	−1.46 (−3.66 to 0.74)	−0.43 (−3.20 to 2.35)	−0.02 (−0.05 to 0.01)	7.73 (2.56 to 12.9)†	−0.18 (−0.51 to 0.14)	−0.14 (−0.66 to 0.37)
Left ventricular mass	−0.06 (−0.11 to –0.01)‡	0.02 (−0.05 to 0.08)	−0.0006 (−0.001 to –0.0000003)	0.014 (−0.10 to 0.13)	−0.02 (−0.03 to –0.01)†	0.02† (0.008 to 0.03)
Prevalent CHD	−3.88 (−11.5 to 3.69)	7.07 (−0.36 to 14.51)	−0.06 (−0.14 to 0.02)	−3.38 (−17.9 to 11.22)	−0.56 (−1.07 to –0.05)‡	0.84 (0.007 to 1.68)‡
Prevalent DM	1.38 (−1.20 to 3.98)	1.72 (−1.50 to 4.94)	−0.008 (−0.04 to 0.02)	3.15 (−2.74 to 9.03)	−0.03 (−0.31 to 0.25)	−0.26 (−0.70 to 0.18)
Ejection fraction	0.07 (−0.04 to 0.18)	0.06 (−0.09 to 0.21)	0.001 (−0.0003 to 0.003)	0.10 (−0.17 to 0.38)	0.0007 (−0.013 to 0.015)	0.011 (−0.011 to 0.03)
Physical activity	0.01 (−0.005 to 0.03)	0.01 (−0.01 to 0.04)	0.00005 (−0.0001 to 0.0003)	−0.02 (−0.06 to 0.02)	−0.002 (−0.004 to 0.0005)	0.003 (−0.0005 to 0.006)
Left atrium dimension	0.05 (−0.12 to 0.21)	0.06 (−0.15 to 0.28)	0.0004 (−0.001 to 0.003)	−0.22 (−0.60 to 0.17)	−0.0003 (−0.022 to 0.021)	0.033 (−0.002 to 0.07)

All presented betas (95% CIs) are based on fully adjusted models.

*Age in this analysis is used as a time-varying covariate.

†P<0.0083 (significant at Bonferroni-corrected p value).

‡P<0.05.

BMI, body mass index; BP, blood pressure; CHD, coronary heart disease; DBP, diastolic blood pressure; DM, diabetes mellitus; HDL, high-density lipoprotein; LVDF, left ventricular diastolic function; SBP, systolic blood pressure.

**Table 3:** Association of risk factors with longitudinal changes in LVDF parameters among men

	E wave	A wave	E/A ratio	DT	Septal e′	E/e′ ratio
Age*	5.38 (2.60 to 8.16)†	23.5 (20.4 to 26.6)†	−0.010 (−0.012 to –0.009)†	0.55 (0.23 to 0.87)†	−0.006 (−0.02 to 0.03)	0.015 (−0.05 to 0.019)
BMI	0.22 (−0.10 to 0.54)	1.25 (0.84 to 1.66)†	−0.007 (−0.01 to –0.003)†	0.86 (0.017 to 1.71)‡	−0.03 (−0.07 to 0.006)	0.12 (0.06 to 0.19)†
SBP	0.14 (0.08 to 0.19)†	0.12 (0.05 to 0.19)†	0.0001 (−0.0007 to 0.0009)	−0.09 (−0.25 to 0.05)	−0.002 (−0.01 to 0.006)	0.028 (0.01 to 0.04)†
DBP	−0.21 (−0.32 to –0,10)†	−0.10 (−0.24 to 0.03)	−0.002 (−0.003 to –0.0004)‡	0.18 (−0.11 to 0.46)	−0.005 (−0.02 to 0.01)	−0.016 (−0.04 to 0.008)
BP-lowering medication	−0.78 (−2.79 to 1.23)	1.90 (−0.66 to 4.47)	−0.02 (−0.05 to 0.005)	2.24 (−3.05 to 7.53)	−0.09 (−0.33 to 0.16)	0.11 (−0.27 to 0.48)
Heart rate	−0.10 (−0.18 to 0.01)	0.21 (0.10 to 0.32)†	−0.003 (−0.004 to –0.002)†	−0.29 (−0.52 to –0.06)‡	0.003 (−0.007 to 0.01)	−0.013 (−0.029 to 0.003)
Total cholesterol	−0.55 (−1.55 to 0.45)	−0.45 (−1.75 to 0.84)	0.013 (−0.0005 to 0.03)	1.42 (−1.23 to 4.06)	−0.12 (−0.25 to 0.02)	0.022 (−0.18 to 0.22)
HDL cholesterol	−0.28 (−3.07 to 2.52)	1.79 (−1.85 to 5.43)	−0.005 (−0.05 to 0.03)	3.03 (−4.41 to 10.47)	0.17 (−0.17 to 0.51)	−0.04 (−0.56 to 0.48)
Lipid-lowering medication	0.23 (−2.11 to 2.58)	−2.19 (−5.20 to 0.83)	0.02 (−0.02 to 0.05)	2.01 (−4.35 to 8.38)	−0.15 (−0.45 to 0.15)	−0.038 (−0.49 to 0.41)
Current smoking	1.32 (−1.53 to 4.16)	2.48 (−1.04 to 6.0)	−0.007 (−0.05 to 0.03)	2.72 (−4.79 to 10.2)	0.04 (−0.39 to 0.47)	−0.08 (−0.73 to 0.57)
Left ventricular mass	−0.08 (−0.14 to –0.03)†	−0.028 (−0.09 to 0.04)	−0.0005 (−0.001 to 0.0002)	0.006 (−0.13 to 0.14)	−0.017 (−0.02 to –0.01)†	0.017 (0.007 to 0.03)†
Prevalent CHD	1.54 (−1.83 to 4.91)	3.1 (−1.40 to 7.54)	0.02 (−0.03 to 0.06)	−1.59 (−10.96 to 7.78)	−0.35 (−0.71 to 0.023)	0.81 (0.26 to 1.37)†
Prevalent DM	1.75 (−1.28 to 4.77)	3.83 (0.06 to 7.60)‡	0.005 (−0.04 to 0.05)	−1.09 (−8.94 to 6.76)	−0.40 (−0.70 to –0.09)‡	0.60 (0.14 to 1.06)‡
Ejection fraction	0.11 (−0.007 to 0.22)	0.04 (−0.11 to 0.18)	0.002 (0.0002 to 0.003)‡	0.28 (−0.023 to 0.59)	0.004 (−0.01 to 0.018)	0.011 (−0.012 to 0.033)
Physical activity	−0.005 (−0.03 to 0.01)	−0.003 (−0.03 to 0.02)	−0.00006 (−0.0004 to 0.0002)	−0.02 (−0.07 to 0.03)	0.002 (−0.0001 to 0.005)	−0.003 (−0.007 to 0.001)
Left atrium dimension	0.11 (−0.09 to 0.30)	−0.21 (−0.46 to 0.04)	0.003 (0.0003 to 0.006)‡	−0.24 (−0.75 to 0.27)	−0.002 (−0.02 to 0.02)	−0.003 (−0.03 to 0.02)

All presented betas (95% CIs) are based on fully adjusted models.

*Age in this analysis is used as a time-varying covariate.

†P<0.0083 (significant at Bonferroni-corrected p value).

‡P<0.05.

BMI, body mass index; BP, blood pressure; CHD, coronary heart disease; DBP, diastolic blood pressure; DM, diabetes mellitus; HDL, high-density lipoprotein; LVDF, left ventricular diastolic function; SBP, systolic blood pressure.

### E wave

Among both women and men, age and SBP were associated with a rise in E wave while DBP and LVM were associated with a decline in E wave over time. Although BMI was associated with a rise in E wave in both sexes, this association was only significant in women (tables 2 and 3 and online supplementary tables 3 and 4).

### A wave

Age, SBP, BMI and HR were associated with a rise in A wave over time in both genders, and DM only in men (tables 2 and 3 and online supplementary tables 3 and 4).

### E/A ratio

Risk factors associated with a decline in E/A ratio were age, DBP and HR in both genders. Only in men, BMI was significantly associated with a decline in E/A ratio and LVEF and LAD with a rise in E/A ratio (tables 2 and 3 and online supplementary tables 3 and 4).

### Deceleration time

Among women, current smoking was the strongest risk factor significantly associated with a rise in DT over time. Age was associated with a rise in DT in both genders. SBP in women and HR in men were significantly associated with a decline in DT. BMI was associated with a rise in DT only in men (tables 2 and 3 (tables 2-3 and online supplementary tables 3 and 4).

### Septal e′

LVM was associated with a decline in septal e′ in both genders. Additionally, LLM and prevalent CHD among women and DM among men were associated with a decline in septal e′. Among women, age and HDL cholesterol were also associated with a rise in septal e′ (tables 2 and 3 and online supplementary tables 3 and 4).

### E/e′ ratio

LVM was associated with a rise in E/e′ ratio in both genders. Additionally, LLM was associated with the rise and HDL cholesterol with a decline in the E/e′ ratio among women. Among men, prevalent CHD, BMI and DM were associated with a rise in the E/e′ ratio (tables 2 and 3 and online supplementary tables 3 and 4). P values for sex interaction in the associations of BMI and DM with the E/e′ ratio were significant.

## Discussion

In the large prospective population-based RS, women had poorer diastolic function than men. However, the tendency of age-related changes in LVDF parameters over time was similar in both genders. Current smoking among women and metabolic factors such as BMI and DM among men were found to be associated with deleterious progression of longitudinal changes in LVDF parameters over time. HDL cholesterol showed a favourable association with LVDF trajectories mainly in women.

Although few studies have shown the intrinsic effect of age and several cardiovascular risk factors on worsening of LVDF parameters,^3 7^ a comprehensive longitudinal assessment of continuous LVDF parameters by gender over time is scant.^6^ Patterns of longitudinal changes in the LVDF indexes over time in our study indicated a progressive impaired relaxation as well as increasing filling pressures with advancing age in both genders. In line with our findings, Kuznetsova *et al* 7 also found a rise in the E/e′ ratio and decline in septal e′ and E/A ratio over time. The LVDF parameters we reported are also comparable to those reported by Caballero *et al* 12 in populations older than 60 years, implying a worsening of diastolic function with ageing.

We found that the postmenopausal women in our study had a worse diastolic function compared with men, providing more evidence regarding the larger burden of diastolic dysfunction among women after menopause.^5 12^ In younger men, a larger decline in most of the LVDF indexes over time was observed. Women have a better diastolic function until 60 years of age after which they experience a steeper decline and worse diastolic function compared with men.^5^ Ageing per se seems to produce more eccentric remodelling and threefold larger apoptosis in men compared with women that might explain a steeper decline in diastolic reserve and the higher prevalence of diastolic dysfunction and HFpEF in women compared with men.^13 14^

Longitudinal analyses of risk factors associated with changes in continuous LVDF parameters over time from a gender-specific perspective are scarce. Kuznetsova *et al*,^7^ based on the risk factors identified in cross-sectional studies, evaluated the longitudinal determinants of LVDF parameters and showed advancing age, higher insulin levels, DBP and HR to worsen LVDF indexes over time. A recent longitudinal analysis of Framingham,^15^ based on categorical LVDF parameters during the 5.6-year follow-up, showed that age, female sex, changes in SBP and DBP, BMI, serum triglycerides and DM were associated with worsening diastolic function in the total population. Our current study expands these findings by examining the risk factors associated with changes in various continuous LVDF parameters over 11 years of follow-up from a gender-specific perspective. The main advantage of analysing the continuous LVDF parameters is a greater power to detect associations and a lower misclassification bias than analysis based on categorical classification.^16^

## Association of risk factors with longitudinal changes in LVDF parameters among women and men

### Blood pressure

SBP and DBP showed significant associations with longitudinal changes in E wave, A wave and E/A ratio among women and men. The opposite direction of the effect for SBP and DBP suggested the effect of pulse pressure (PP). Accordingly, when we substituted SBP and DBP with PP in our analyses, PP was significantly associated with changes in these parameters among women and men. In several epidemiological studies, PP has shown a superior predictive value compared with SBP or DBP alone.^17 18^ Higher PP is associated with elevated stress of the left ventricle which can result in ventricular hypertrophy and failure, critical determinants of LVDF.^18^

### Metabolic factors

Previous cross-sectional studies have independently associated diastolic dysfunction with BMI and DM.^19^ In our study, DM was found to be strongly associated with worsening of LVDF parameters in men. Expanded myocardial fibrosis as well as accelerated apoptosis are among the pathophysiological features of diabetic cardiomyopathy.^20^ While several previous studies have shown larger deterioration of LVDF among diabetics,^8^ data regarding sex differences in the association of DM on LVDF are scarce and conflicting. Diabetes was found to be an independent contributor to LVM among women in the Framingham Heart Study^21^ but among both women and men from the Cardiovascular Health Study^22^ and the Strong Heart Study.^23^

In our study, a larger association of BMI with worsening of LVDF over time was found among men than in women. The only prior, cross-sectional, study that evaluated sex differences of obesity on LVDF reported no association between BMI and LVDF indexes in women >65 years but did describe an association between septal e′ and abdominal adiposity among younger women. Among men, BMI and abdominal obesity were associated with a higher likelihood of diastolic dysfunction.^24^ The obesity-related mechanisms might be different for women and men. While for younger women, the effect of obesity might act through its influence on SBP, the effect seems to be predominantly direct for men >65 years.^24^

### Smoking and lipid profile

Current smoking was only associated with a rise in DT among women in our study. Smoking commonly precedes the development of HFpEF.^25^ While smoking confers a greater CHD risk in women compared with men,^26^ sex differences in the setting of HF have not been reported.^27^ Smoking has been suggested to significantly affect LVDF independently of its role as a risk factor for coronary atherosclerosis and through other independent pathways.^28^

We found a favourable association of HDL cholesterol with diastolic function over time among women. Moreover, the use of lipid-lowering medication, as a proxy for chronic dyslipidaemia, was associated with worse LVDF over time. Previous cross-sectional studies have associated hyperlipidaemia with coronary endothelial dysfunction and with myocardial damage independent of ischaemia, leading to diastolic dysfunction.^29^ Low levels of HDL cholesterol and elevated levels of total cholesterol are known risk factors for CHD and increasing LVM, both important factors leading to diastolic dysfunction. While increasing HDL levels have a more favourable effect in women compared with men, such gender differences in the association of HDL with LVDF require further study.

### Study strengths and limitations

Our study was based on a large group of women and men from a population-based cohort with repeated echocardiographic examinations over 11 years of follow-up. The longitudinal design allowed the use of linear mixed effect model to analyse progressive long-term alterations in continuous LVDF parameters. Availability of the well-defined set of covariates and detailed characterisation of the cohort allowed to examine LVDF parameters and their correlates from a gender-specific perspective. Nevertheless, limitations of our study also merit consideration. The gold standard for diastolic function measurement is the pressure–volume relationship which is an invasive approach. However, Doppler measurements of mitral inflow and TDI allows for a valid non-invasive measurement of diastolic function.^10 30^ Echocardiography has proven to be a useful tool for assessing diastolic function, in order to minimise inherent limitations operator-dependent, a standardised protocol was used by four trained echocardiographers with good inter-reader and intra-reader agreement.^11^ Our population included individuals of European ancestry. Therefore, the generalisability of our findings to other ethnicities should be performed with caution. As inherited to all longitudinal cohort studies, survival bias cannot be entirely ruled out.

## Conclusions

In our large population-based study, women were found to have poorer diastolic function than men. However, age-related changes in continuous LVDF parameters were comparable in both genders. Our findings highlight the correlates of asymptomatic diastolic dysfunction among women and men. The differential association of risk factors with LVDF among women and men could provide further hypothesis regarding the transition from a healthy heart to the development of HFpEF.^5^

**Key messages**

**What is already known on this subject?**

- Left ventricular diastolic dysfunction and heart failure with preserved ejection fraction occurs more frequent in women than men, but it is not clear what risk factors are associated with these gender differences.

**What might this study add?**

- Our results show the differential association of risk factors with longitudinal alterations in the left ventricular diastolic function (LVDF) parameters among women and men. We observed a larger deleterious association for smoking among women and for body mass index and diabetes  mellitus among men with longitudinal changes in LVDF parameters over time. The favourable association of high-density lipoprotein cholesterol with LVDF was more pronounced among women.

**How might this impact on clinical practice?**

- Changes over time in the trajectories of continuous LVDF parameters among women and men and their associated risk factors provide a novel hypothesis platform for further medical research.
