# Supplementary material for: Risk factors for longitudinal changes in left ventricular diastolic function among women and men
Source: Heart. 2019 Apr 1;105(18):1414–22. doi: 10.1136/heartjnl-2018-314487 (PMC6820149; doi:10.1136/heartjnl-2018-314487)
Supplement: Supplementary file 1 [file heartjnl-2018-314487supp001.docx]

**SUPPLEMENTAL MATERIAL**

Rueda-Ochoa OL, et al. Risk factors for longitudinal changes in left ventricular diastolic function among women and men: the Rotterdam Study.

**Supplemental Methods**

Echocardiography

Assessment of Cardiovascular Risk Factors

Statistical Analysis

**Supplemental Results**

Non-returning participants

Online Table 1. Baseline clinical and echocardiographic characteristics of the participants for the analysis of two left ventricular diastolic function parameters.

Online Table 2. Clinical and echocardiographic characteristics at the first examination for the individuals that participated only at the first examination and did not return for the two follow-up examinations.

Online Table 3. Association of risk factors with longitudinal changes in left ventricular diastolic function parameters among women.

Online Table 4. Association of risk factors with longitudinal changes in left ventricular diastolic function parameters among men.

Online Table 5. Left ventricular diastolic function parameters stratified by age and gender.

Online Figure 1. Flow chart for the participants included in the analysis of two left ventricular diastolic function parameters measured two times

**References**

**Supplemental Methods**

**Echocardiography:**

For each participant, one echocardiogram was obtained at each examination. In the first examination, the first 40% of the echocardiograms were performed with a commercially available ultrasonography system (AU3 Partner, Esaote Biomedica, with a 3.5/2.5 MHz transducer) and the followings with Acuson Cypress, with a 3V2c transducer. For the subsequent second and third examinations, a standardized protocol was used which also included two-dimensional resting transthoracic echocardiography performed by experienced echocardiographers with an identical standardized protocol for all participants and a commercially available ultrasonography system (Vivid I, GE Healthcare, Little Chalfont, UK), with a 2.5 MHz transducer. All examinations were performed by the same echocardiographers using the same protocol. As described previously, inter-reader and intra-reader agreements were good(1). All images were digitally stored and assessed offline by the echocardiographers.

The protocol included 2-dimensional scanning in the parasternal long and short axis views, the apical and subcostal views. In addition, 2-dimension guided M-mode measurements of left ventricle were obtained by scanning in the parasternal long axis view.

Left atrial diameter (LAD), left ventricular end diastolic diameter (LVEDD), left ventricular end systolic diameter (LVESD), interventricular septum thickness (IVST), left ventricular posterior wall thickness (LVPWT) and left ventricular ejection fraction were the left-sided measurements. Relative wall thickness was calculated according to the formula (2 * LVPWT)/(LVEDD) (2). Left ventricular mass (LVmass) in grams was calculated according to the formula by Devereux and colleagues as 0.8 * (1.04 * ((LVEDD + IVST + LVPWT)3 - LVEDD3)) + 0.6(3), and was indexed with Body Surface Area (BSA) (2). Left ventricular fractional shortening (FS) was calculated using the formula: FS = (LVEDD-LVESD)/LVEDD * 100%(4).

**Assessment of Cardiovascular Risk Factors:**

Medical history, current health status, smoking and use of medications were assessed by a trained interviewer at the home visit using a computerized questionnaire. Body mass index (BMI) was calculated as weight in kilograms divided by height in squared meters. Systolic blood pressure (SBP) and diastolic blood pressure (DBP) were measured twice in sitting position at the right upper arm. We used the average of two consecutive measurements. Hypertension was defined as SBP >140 mm Hg, DBP >90 mm Hg, or use of blood pressure-lowering medication with an indication for hypertension. Heart rate (HR) was measured with an oximeter in the second finger of the right hand and the average of two consecutive measurements was used. Total and high density lipoprotein (HDL) cholesterol and glucose levels were measured with the use of standardized laboratory techniques. Diabetes mellitus (DM) was defined as fasting glucose >6.9 mmol/L, nonfasting glucose >11.0 mmol/L, use of blood glucose-lowering medication, or a previous diagnosis of DM. A history of coronary heart disease (CHD) was defined as a myocardial infarction or coronary revascularization procedure (5, 6). Physical activity was evaluated using LASA questionnaire (LAPAQ) and accelerometer (Actiwatch) (7).

**Statistical Analysis:**

For the analysis of the diastolic dysfunction indices, using each of the parameters as continuous variables, distribution of the outcome variable was graphically assessed for normality (histograms, box plots, and QQ plots). To select the correct function for the variable age (as a time-varying covariate), several different initial models including linear and non-linear functions together with interaction terms for age with other covariates were built. Outlier values were removed. The following covariates were included in the fixed part of the linear mixed models: Age (as time-varying covariate), systolic and diastolic blood pressure (SBP, DBP), heart rate (HR), total and high-density lipoprotein (HDL) cholesterol, blood pressure and lipid lowering medications, diabetes mellitus (DM), current smoking, previous coronary heart disease (CHD), left ventricular mass indexed by body surface area (LVM), left ventricular ejection fraction (LVEF), physical activity, left atrial diameter (LAD) and cohort. In the random part of the linear mixed model, age was the only variable (as a time-varying covariate) included. First, we evaluated a full model, including interactions terms, comparing model with random intercept vs model with both intercept and slope random. Second, we evaluated the linear and non-linear terms in the random part of the model and selected the model with lower AIC. Third, we evaluated the fixed part of the model, comparing full model with interactions terms vs model without interactions terms and selected the model with lower AIC. Finally, we evaluated the linear and non-linear terms in the fixed part of the model and selected the model with lower AIC. Non-linear terms evaluated were polynomials, and natural splines quadratic and cubic. Convergence problems of some models were solved increasing the mathematical iterations, using optimizer (bobyqua optimizer) and centralized continuous variables if it was needed.

A residual analysis was made to all final models. Several covariates were missing in <5% of the participants and were imputed using fully conditional specification (Markov chain Monte Carlo method) with a maximum iteration number of five.

**Supplemental Results**

**Non-returning participants:**

From the 3,420 participants who were present at examination 1 but not at the follow-up examinations, 1,867 had died before the next follow-up visit. Of the 3,422 surviving participants, 1,553 did not return for the follow-up examinations. Survivors who did not return were older; more often women, hypertensive, current smoker, and diabetic; and had higher mean values for BMI, SBP, and HR. Among the echocardiographic parameters, the non-returning participants had larger LVM and left atrial (LA) diameter, larger chamber dimensions, higher relative wall thickness (RWT), smaller FS, higher A wave and DT, and lower E/A ratio. (Online Table 5)

**Online Table 1.** Baseline clinical and echocardiographic characteristics of the participants for the analysis of two left ventricular diastolic function parameters.

|  | **WOMEN (n=882)** | **MEN**  **(n=646)** | **p-value*** |
| --- | --- | --- | --- |
| **Clinical Features** |  |  |  |
| Age, years | 73.63 (4.98) | 73.64 (4.95) | 0.990 |
| BMI, kg/m² | 27.27 (4.22) | 27.02 (3.06) | 0.201 |
| SBP, mmHg | 150.82 (20.86) | 152.35 (20.13) | 0.151 |
| DBP, mmHg | 85.50 (11.00) | 85.81(10.98) | 0.594 |
| Blood pressure Lowering Medication, n (%) | 414 (46.94) | 338 (52.32) | 0.038 |
| Hypertension, n (%) | 764 (86.6) | 583 (90.3) | 0.027 |
| Heart Rate, beats/min | 68.15 (9.46) | 65.21 (10.64) | <0.001 |
| Total Cholesterol, mmol/L | 5.73 (1.04) | 5.06 (1.04) | <0.001 |
| HDL-cholesterol, mmol/L | 1.64 (0.42) | 1.34 (0.34) | <0.001 |
| Lipid Lowering Medication, n (%) | 232 (26.3) | 236 (36.5) | <0.001 |
| Current Smoker, n (%) | 83 ( 9.4) | 43 ( 6.7) | 0.048 |
| Prevalent CHD, n (%) | 35 ( 3.97) | 99 (15.3) | <0.001 |
| Prevalent DM, n (%) | 137 (15.5) | 108 (16.7) | 0.531 |
| **Echocardiography Features** |  |  |  |
| LVM index, g/m² | 66.96 (14.80) | 74.47(19.38) | <0.001 |
| Left Atrium Diameter/BSA†, mm/m² | 22.79 (2.93) | 22.18 (2.83) | <0.001 |
| LVEDD, mm | 49.36 (4.29) | 53.23 (5.07) | <0.001 |
| LVESD, mm | 28.31 (3.50) | 31.35 (4.97) | <0.001 |
| Relative Wall Thickness, cm | 0.27 (0.05) | 0.27 (0.05) | 1.0 |
| Fractional Shortening, % | 43.14 (5.99) | 41.92 (7.80) | <0.001 |

* p-value for comparison of different characteristics between men and women.

Values are mean (± standard deviation) or numbers (percentages).

BMI: Body mass index, BSA: Body surface area, CHD: coronary heart disease, DM: Type 2 diabetes mellitus, DBP: Diastolic blood pressure, LVEDD: Left ventricle end diastolic dimension, LVESD: Left ventricle end systolic dimension, LVM: Left ventricular mass, SBP: Systolic blood pressure.

**Online Table 2.** Left ventricular diastolic function parameters stratified by age and gender.

|  | **55 – 64 years old** | | | **65 – 74 years old** | | | **>= 75 years old** | | |
| --- | --- | --- | --- | --- | --- | --- | --- | --- | --- |
|  | **Women** | **Men** | **p-value*** | **Women** | **Men** | **p-value*** | **Women** | **Men** | **p-value*** |
| **E wave, cm/s** | N:276  68  (13.08) | N:194  65.94  (12.41) | 0.0867 | N: 1253  67.37  (13.0) | N: 934  64.03  (12.8) | <0.001 | N: 946  67.14  (13.19) | N: 693  64.6  (13.69) | <0.001 |
| **A wave, cm/s** | N: 291  73.07  (13.27) | N: 201  68.83  (13.19) | <0.001 | N: 1332  80.79  (17.04) | N: 960  73.53  (16.42) | <0.001 | N:1013  89.86  (17.9) | N:711  82.2  (18.51) | <0.001 |
| **E/A ratio** | N:274  0.947  (0.195) | N: 183  0.97  (0.20) | 0.22 | N: 1259  0.854  (0.175) | N: 875  0.88  (0.19) | 0.0013 | N:954  0.77  (0.17) | N: 658  0.80  (0.19) | <0.001 |
| **Deceleration Time, msec** | N: 289  199  (33.25) | N: 207  201.86  (37.8) | 0.365 | N:1325  204.01  (33.83) | N:973  207.5  (38.48) | 0.021 | N: 1018  207.23  (39.24) | N: 714  213.67  (42.33) | 0.0012 |
| **Septal e’, cm/s** | N/A† | N/A† | N/A† | N: 863  7.22  (1.78) | N:651  7.52  (1.67) | <0.001 | N:981  6.5  (1.73) | N:713  6.99  (1.87) | <0.001 |
| **E/e’ ratio** | N/A† | N/A† | N/A† | N:799  10.06  (2.5) | N: 610  9.28  (2.41) | <0.001 | N: 884  10.86  (2.73) | N:638  9.89  (2.64) | <0.001 |

Values are mean (± standard deviation).

* p-value for comparison of different values of left ventricular diastolic function parameters for women and men in each age group.

† N/A indicates that e’ and E/e’ ratio were not available at the indicated examination.

**Online Table 3.** Association of risk factors with longitudinal changes in left ventricular diastolic function parameters among women.

| **WOMEN** | **E wave** | **A wave** | **E/A** | **DT** | **e`septal** | **E/e`** |
| --- | --- | --- | --- | --- | --- | --- |
|  | **MIXED** | **MIXED** | **MIXED** | **MIXED** | **MIXED** | **MIXED** |
| Agetime | † | † | † | † | ‡ |  |
| BMI | ‡ | † |  |  |  |  |
| SBP | † | † |  | ‡ |  |  |
| DBP | † |  | † |  |  |  |
| BP lowering medication |  |  |  |  |  |  |
| Heart rate |  | † | † |  |  |  |
| Total Cholesterol |  |  |  |  |  |  |
| HDL Cholesterol |  |  |  |  | † | ‡ |
| Lipid low medication |  |  |  |  | ‡ | † |
| Current SMK |  |  |  | † |  |  |
| LVM | ‡ |  |  |  | † | † |
| Prevalent CHD |  |  |  |  | ‡ | ‡ |
| Prevalent DM |  |  |  |  |  |  |
| Ejection fraction |  |  |  |  |  |  |
| Physical activity |  |  |  |  |  |  |
| LAD |  |  |  |  |  |  |

*Age in this analysis is used as a time-varying covariate. †P< 0.0083 (significant at Bonferroni
 corrected P value); ‡P<0.05.

BMI: Body mass index, BP: blood pressure, CHD: Coronary heart disease, DBP: Diastolic blood pressure,
 DM: Diabetes Mellitus, HDL: High density lipoprotein, SBP: Systolic blood pressure.

Values are betas (95% confidence intervals). All presented betas (95% confidence intervals) are based on
 fully adjusted models

Sex-specific differences are highlighted in yellow in the table.

**Online Table 4.** Association of risk factors with longitudinal changes in left ventricular diastolic function parameters among men.

| **MEN** | **E wave** | **A wave** | **E/A** | **DT** | **e`septal** | **E/e`** |
| --- | --- | --- | --- | --- | --- | --- |
|  | **MIXED** | **MIXED** | **MIXED** | **MIXED** | **MIXED** | **MIXED** |
| Agetime | † | † | † | † |  |  |
| BMI |  | † | † | ‡ |  | † |
| SBP | † | † |  |  |  | † |
| DBP | † |  | ‡ |  |  |  |
| BP lowering medication |  |  |  |  |  |  |
| Heart rate | ‡ | † | † | ‡ |  |  |
| Total Cholesterol |  |  |  |  |  |  |
| HDL Cholesterol |  |  |  |  |  |  |
| Lipid low medication |  |  |  |  |  |  |
| Current SMK |  |  |  |  |  |  |
| LVM | † |  |  |  | † | † |
| Prevalent CHD |  |  |  |  |  | † |
| Prevalent DM |  | ‡ |  |  | ‡ | ‡ |
| Ejection fraction |  |  | ‡ |  |  |  |
| Physical activity |  |  |  |  |  |  |
| LAD |  |  | ‡ |  |  |  |

*Age in this analysis is used as a time-varying covariate. †P< 0.0083 (significant at Bonferroni
 corrected P value); ‡P<0.05.

BMI: Body mass index, BP: blood pressure, CHD: Coronary heart disease, DBP: Diastolic blood pressure,
 DM: Diabetes Mellitus, HDL: High density lipoprotein, SBP: Systolic blood pressure.

Values are betas (95% confidence intervals). All presented betas (95% confidence intervals) are based on
 fully adjusted models

Sex-specific differences are highlighted in yellow in the table.

**Online Table 5.** Clinical and echocardiographic characteristics at the first examination for the individuals that participated only at the first examination and did not return for the two follow-up examinations.

|  | **Participants (n=1,619)** | **Non-Returning Individuals (n=1,553)** | **p-value*** |
| --- | --- | --- | --- |
| **Clinical Features** |  |  |  |
| Age, years | 67.45 (5.03) | 71.47 (6.33) | <0.001 |
| Female Sex, n (%) | 931 (57.5) | 1016 (65.4) | <0.001 |
| BMI, kg/m² | 27.37 (3.72) | 27.88 (4.17) | <0.001 |
| SBP, mmHg | 144.70 (18.90) | 150.16 (20.54) | <0.001 |
| DBP, mmHg | 80.85 (10.08) | 80.18 (10.32) | 0.065 |
| Blood Pressure Lowering Medication, n (%) | 531 (33.1) | 708 (46.2) | <0.001 |
| Hypertension, n (%) | 1055 (65.9) | 1140 (74.7) | <0.001 |
| Heart Rate, beats/min | 67.85 (10.27) | 69.34 (10.93) | <0.001 |
| Total Cholesterol, mmol/L | 5.73 (0.98) | 5.71 (0.96) | 0.562 |
| HDL-cholesterol, mmol/L | 1.47 (0.39) | 1.49 (0.40) | 0.154 |
| Lipid Lowering Medication, n (%) | 343 (21.4) | 355 (23.2) | 0.568 |
| Current Smoker, n (%) | 166 (10.5) | 207 (13.6) | 0.363 |
| Prevalent CHD, n (%) | 91 ( 5.6) | 106 ( 6.8) | 0.728 |
| Prevalent DM, n (%) | 160 ( 9.9) | 201 (12.9) | 0.376 |
| **Echocardiography Features**† |  |  |  |
| LVM index, g/m² | 72.29 (17.33) | 74.35 (18.19) | 0.013 |
| Left Atrium Diameter/BSA‡, mm/m² | 21.13 (2.61) | 21.56 (2.99) | <0.001 |
| LVEDD, mm | 51.08 (5.03) | 50.91 (5.28) | 0.353 |
| LVESD, mm | 30.71 (4.76) | 31.11 (5.30) | 0.025 |
| Relative Wall Thickness, cm | 0.29 (0.05) | 0.30 (0.06) | <0.001 |
| Fractional Shortening, % | 38.52 (14.54) | 36.50 (16.26) | <0.001 |
| E wave, cm/s | 65.53 (14.56) | 64.97 (15.74) | 0.305 |
| A wave, cm/s | 73.26 (15.58) | 77.32 (16.73) | <0.001 |
| Deceleration Time, msec | 207.86 (40.43) | 214.15 (46.86) | <0.001 |
| E/A ratio | 0.93 (0.23) | 0.88 (0.36) | <0.001 |

*p-value for comparison of different characteristics between participants and non-returning individuals.

† In the first evaluation measurements of Echo TDI were not available.

Values are mean (± standard deviation) or numbers (percentages).

BMI: Body mass index, BSA: Body surface area, CHD: coronary heart disease, DM: Type 2 diabetes mellitus, DBP: Diastolic blood pressure, LVEDD: Left ventricle end diastolic dimension, LVESD: Left ventricle end systolic dimension, LVM: Left ventricular mass, SBP: Systolic blood pressure.

**Online Figure 1.** Flow chart for the participants included in the analysis of two left ventricular diastolic function parameters measured two times.

First Examination

(2002-2005)

N=5,289

n=5,289

Second Examination

(2009-2012)

N=3,366

n=5,289

Third Examination

(2014-2016)

N=2,013

n=5,289

Present in 2 examinations

N=1,925

n=5,289

Included in the analysis

N= 1,528

n=5,289

Excluded due to:

- Poor echo images
 - AF or pacemaker

- Valvular disease

- Heart failure

N= 397

**References**

1. Kardys I, Deckers JW, Stricker BH, Vletter WB, Hofman A, Witteman JC. Echocardiographic parameters and all-cause mortality: the Rotterdam Study. Int J Cardiol. 2009;133(2):198-204.

2. Lang RM, Badano LP, Mor-Avi V, Afilalo J, Armstrong A, Ernande L, et al. Recommendations for cardiac chamber quantification by echocardiography in adults: an update from the American Society of Echocardiography and the European Association of Cardiovascular Imaging. J Am Soc Echocardiogr. 2015;28(1):1-39 e14.

3. Devereux RB, Alonso DR, Lutas EM, Gottlieb GJ, Campo E, Sachs I, et al. Echocardiographic assessment of left ventricular hypertrophy: comparison to necropsy findings. Am J Cardiol. 1986;57(6):450-8.

4. Schiller NB, Shah PM, Crawford M, DeMaria A, Devereux R, Feigenbaum H, et al. Recommendations for quantitation of the left ventricle by two-dimensional echocardiography. American Society of Echocardiography Committee on Standards, Subcommittee on Quantitation of Two-Dimensional Echocardiograms. J Am Soc Echocardiogr. 1989;2(5):358-67.

5. Leening MJ, Kavousi M, Heeringa J, van Rooij FJ, Verkroost-van Heemst J, Deckers JW, et al. Methods of data collection and definitions of cardiac outcomes in the Rotterdam Study. Eur J Epidemiol. 2012;27(3):173-85.

6. Hofman A, Brusselle GG, Darwish Murad S, van Duijn CM, Franco OH, Goedegebure A, et al. The Rotterdam Study: 2016 objectives and design update. Eur J Epidemiol. 2015;30(8):661-708.

7. Nagueh SF, Appleton CP, Gillebert TC, Marino PN, Oh JK, Smiseth OA, et al. Recommendations for the evaluation of left ventricular diastolic function by echocardiography. J Am Soc Echocardiogr. 2009;22(2):107-33.
